# Chronic postsurgical pain (CPSP): an underestimated problem after incisional hernia treatment

**DOI:** 10.1007/s10029-024-03027-7

**Published:** 2024-03-25

**Authors:** A. Widder, L. Reese, J. F. Lock, A. Wiegering, C.-T. Germer, H. L. Rittner, U. A. Dietz, N. Schlegel, M. Meir

**Affiliations:** 1 Department of General, Visceral, Transplantation, Vascular and Paediatric Surgery, Centre of Operative Medicine (ZOM), University Hospital of Wuerzburg, Würzburg, Germany; 2 Department of Anesthesiology, Intensive Care, Emergency and Pain Medicine, University Hospital Wuerzburg, Würzburg, Germany; 3 Department of Anesthesiology, Intensive Care, Emergency and Pain Medicine, Centre for Interdisciplinary Pain Medicine, University Hospital of Wuerzburg, Würzburg, Germany; 4 Department of Visceral, Vascular and Thoracic Surgery, Cantonal Hospital Olten (soH), Olten, Switzerland

**Keywords:** Open hernia repair, Chronic postsurgical pain, Incisional hernia, Analgesics score

## Abstract

**Background:**

Chronic postsurgical pain (CPSP) is a potential long-term problem following open incisional hernia repair which may affect the quality of life of patients despite successful anatomical repair of the hernia. The aim of this manuscript was to identify the incidence and outcome of patients following open incisional hernia repair in respect of risk factors to develop CPSP.

**Methods:**

A single-center retrospective analysis of patients who underwent open incisional hernia repair between 2015 and 2021 was performed. Pre-existing conditions (e.g., diabetes mellitus and malignancy), hernia complexity, postoperative complications, and postoperative pain medication were analyzed using the local database. Quality of life and CPSP were assessed using the EuraHS Quality of Life (QoL) questionnaire.

**Results:**

A total of 182 cases were retrospectively included in a detailed analysis based on the complete EuraHS (QoL) questionnaire. During the average follow-up period of 46 months, this long-term follow-up revealed a 54.4% incidence of CPSP and including a rate of 14.8% for severe CPSP (sCPSP) after open incisional hernia surgery. The complexity of the hernia and the demographic variables were not different between the group with and without CPSP. Patients with CPSP reported significantly reduced QoL. The analgesics score which includes the need of pain medication in the initial days after surgery was significantly higher in patients with CPSP than in those without (no CPSP: 2.86 vs. CPSP: 3.35; *p* = 0.047).

**Conclusion:**

The presence of CPSP after open incisional hernia repair represents a frequent and underestimated long-term problem which has been not been recognized to this extent before. CPSP impairs QoL in these patients. Patients at risk to develop CPSP can be identified in the perioperative setting by the need of high doses of pain medication using the analgesics score. Possibly timely adjustment of pain medication, even in the domestic setting, could alleviate the chronicity or severity of CPSP.

## Introduction

With an incidence of 3–20% incisional hernias occur frequently after open abdominal surgery [1–3]. Previously certain risk factors including wound infection, obesity, diabetes mellitus, oral (anti)coagulants, age, male, malignancy, and anemia were identified to contribute to the development of incisional hernias. Symptomatic incisional hernia usually represents an indication for surgical intervention which has the aim to achieve an anatomical repair of the hernia. Patients who suffer from incisional hernia suffer from pain, discomfort, or restrictions in everyday life or work [4].

However, after surgical treatment of incisional hernia which includes the successful anatomical repair of the abdominal wall, long-term chronic pain may occur. Chronic postsurgical pain (CPSP) is defined as pain [≥ 3 in a numeric rating scale (NRS) from 0 (no pain) to 10 (worst pain imaginable)] lasting at least 3 months postoperatively that interferes with daily activities, is perceived as bothersome, and sometimes has neuropathic components [5]. This pain relates to the surgical region and is described as either more severe than preoperatively or new. However, CPSP after open incisional hernia repair is poorly studied. Previous studies have described a chronic pain rate after laparoscopic hernia repair of 26.2–39% [4, 6–9]. The incidence of chronic pain after general visceral surgery is thought to be between 5 and 85% [10, 11]. In a randomized, controlled study, it was shown that approx. 10% of patients continue to have complaints after such an operation and are not satisfied with the result [12]. One reason for this dissatisfaction is the occurrence of postoperative pain.

Whether the postsurgical pain is related to a preoperatively existing symptom complex has not yet been determined. In many postoperative evaluations following hernia repair, most patients express discomfort due to the pain and the resulting restrictions in everyday life and leisure time. Furthermore, sufficient therapy of CPSP is challenging. In the absence of incisional hernia recurrence, patients are treated for pain with analgesic medication and physical therapies. In a few cases, surgical interventions to replace the mesh or remove fixation sutures have been successful [3]. However, this means a second surgery, which also is associated with new risks.

To offer a more targeted therapy for CPSP in the future, risk factors and indicators remain to be identified to minimize or even prevent chronic pain by optimized perioperative analgesia. In a prospective international, multi-institutional study, risk factors including strong preoperative pain, young age, female gender have already been identified [13]. However, the overall incidence of CPSP in a larger patient cohort is unknown and it is unclear whether increased perioperative analgesic medication to relieve perioperative pain is itself a risk factor or whether appropriate perioperative pain management reduces the risk of developing CPSP. Therefore, we performed a detailed retrospective analysis of patients after open incisional hernia repair, focusing specifically on the overall incidence of CPSP, risk factors, and perioperative pain management including the potentially predictive value of our previously established analgesics score [14].

## Materials and methods

### Study design

All patients who underwent open surgical care for an incisional hernia at the Department of Surgery at the University Hospital between January 1, 2015, and December 31, 2021 were screened for possible data analysis. Only patients after incisional hernia repair with retromuscular (sublay) mesh placement were included in our analysis. All patients who had completed an EuraHS Quality of Life (QoL) questionnaire and gave informed consent were included in the retrospective single-center data analysis. The study was approved by the local ethics committee (No. 109/21).

### Data acquisition

From the local database, clinical data were retrospectively obtained.

- Baseline patients’ characteristics: age at operation, gender, Charlson Comorbidity Index (CCI)—a method for classifying comorbidities that can change the risk of death [15].
- Comorbidities of patients: diabetes mellitus, obesity, anticoagulation with oral anticoagulants and/or platelet aggregation inhibitors, active smoking, therapy on immunosuppressant medication and/or steroids, and tumor [8].
- Surgery-related items: duration of surgery, type of mesh (heavy weight mesh or light-weight mesh), transfascial fixation of the mesh, complications according to the Clavien–Dindo classification a tool to standardize the reporting of surgical outcomes [16], hemoglobin difference (preoperative versus at discharge), and length of hospital stay of the patients.

Another investigational variable was the complexity of the hernia. By classifying the complexity of incisional hernias, non-complex hernias with a smaller surgical extent and complex hernias with a correspondingly larger surgical extent can be better compared. Complex incisional hernia were defined having defects with a width > 10 cm [17].

A retrospective survey using the EuraHS QoL questionnaire [18, 19] was administered to all patients. In our study, the questionnaire is based on eight questions for three dimensions, which are answered using a numerical rating scale (0–10):

- pain after incisional hernia repair (3 questions).
- limitation in daily life as well as activities and work (4 individual questions).
- cosmetic perception after surgery (1 question).

For a better overview, we created individual variables as the sum of the NSR given for all questions of one dimension.

For the first dimension of the questionnaire, we defined a variable called “CPSP score” the sum of the NSR values given for the first three questions about pain at rest, during movement and the strongest pain of the past weeks of the EuraHS QoL questionnaire. A minimum of 0 and a maximum of 30 points could be achieved for the “CPSP score”.

For the second dimension of the questionnaire, we defined the sum of four questions as “daily-life score”. The limitations in daily life was asked in four questions: disability in the household/at work, during grocery shopping, in sports and during heavy physical work. A minimum of 0 and a maximum of 40 points could be achieved for the “daily-life score”.

The third dimension is listed as the variable “cosmetic outcome”. One question was asked about the esthetic perception of the scar/the shape of the abdomen. A minimum of 0 and a maximum of 10 points could be achieved for the “cosmetic outcome”.

The quality of life is the sum of all questions in the questionnaire as defined by the authors [18]. A minimum of 0 and a maximum of 80 points could be achieved for the “quality of life”.

### Definition of CPSP in this study

We followed the ICD-11 definition of chronic postsurgical pain (CPSP) is a pain that persists beyond the healing process and lasts for at least three months [10]. In our study, we defined chronic postsurgical pain (CPSP) in the present EuraHS QoL questionnaire as a score ≥ 3 on the numerical rating scale (NRS) of 0–10 in one of the three questions of the first dimension( 0 representing no pain and 10 representing maximal pain) [18].

In addition, we defined moderate (modCPSP) as pain between ≥ 3 and ≤ 6 and severe CPSP (sCPSP) as pain > 6 on the NRS in one of the three questions in the first dimension of the EuraHS QoL questionnaire [20].

### Analgesics score

We used the analgesics score previously established in CPIP to enable comparison of postsurgical pain [14]. The analgesics score is based on the need for postoperative pain medication on the day of discharge to achieve freedom from pain. To quantify pain postoperatively, the analgesics taken at the timepoint of hospital discharge were defined according to their potency using an ascending point scale based on the WHO analgesics ladder and the dose taken. With this scale, an analgesic score could be calculated. Each substance (amount) was assigned a scoring. To avoid bias due to analgesics and co-analgesics already taken preoperatively, these were deducted from the total score according to their Analgesics Score Scale point value. The analgesics score is the sum of all analgesic medications at hospital discharge. Postoperative analgesic therapy was adjusted based on standard procedures until the patients were pain free at rest (NRS < 3). If a patient was not pain free, the analgesia was adjusted, this was documented daily during the hospital stay. NSAIDs (paracetamol, metamizol, ibuprofen) and opioids (tilidine, palladone) are used as standard for pain management. Co-analgesics such as amitriptyline, pregabalin, or lorazepam are rarely used for neuropathic pain.

### Statistical analysis

Statistical analysis was performed using IBM SPSS 28.0 (IBM SPSS, Armonk, New York, USA). Differences between groups were calculated using Welch’s test and *χ*^2^-test as well as single factor variance and analysis of covariance or as repeated measure ANOVA. In the case of multiple *T* tests, a post hoc test was used by means of Bonferroni correction to detect individual comparisons between the groups without risking an alpha error. Significance was set at *p* < 0.05. Descriptive analyses included mean (MV), minimum (min), and maximum (max) values given as range, percentage, and standard deviation.

## Results

### Characterization of the study population

**Fig. 1:**
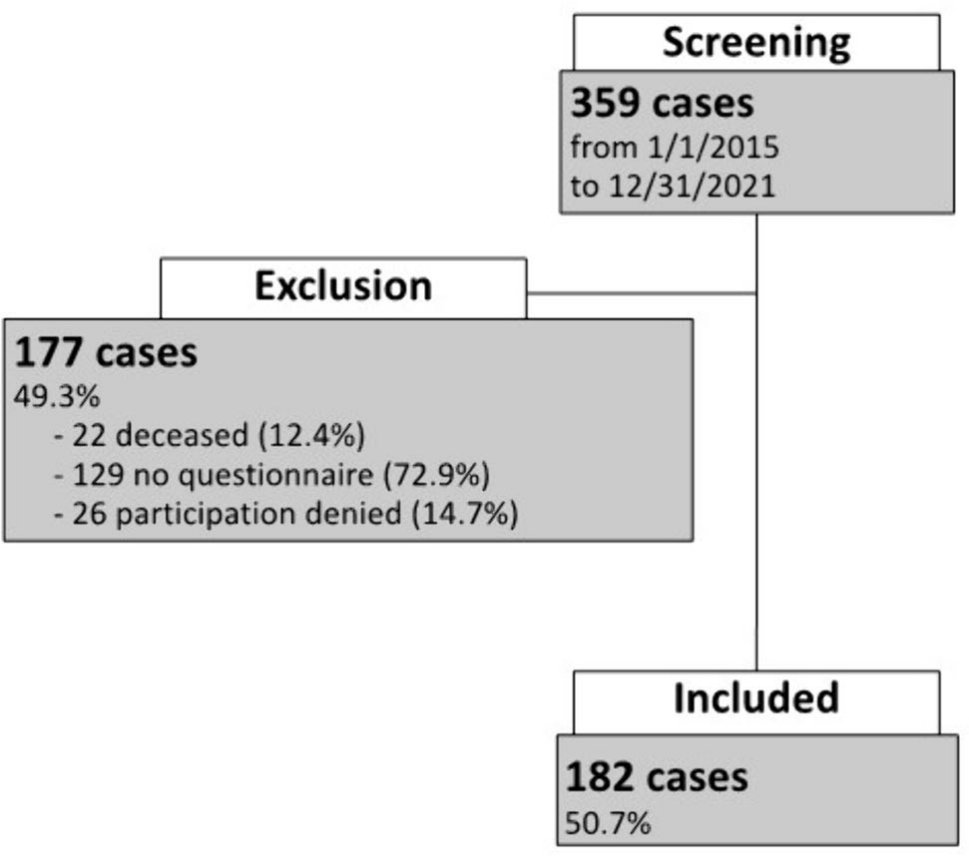
Study population

359 patient cases in which open incisional hernia repair was performed were identified to be eligible for this study. As shown in Fig. 1, 177 (49.3%) patient cases had to be excluded from the data analysis due to the following reasons: 22 patients were deceased, 26 patients refused to participate in the study, and 129 cases were excluded due to missing contact information. Finally, a total of 182 patients with a complete EuraHS QoL questionnaire were included in this study and analyzed. The mean follow-up was 46.6 months with a range of 20–101 months. Overall, the study population had a mean age of 59.5 years and a CCI of 3.41 with a calculated average 1-year mortality of 52% [15]. 33.5% of all patients had a history of malignancy and three patients were operated due to a recurrent hernia. In addition, other risk factors for the development of an incisional hernia were present as revealed by a rate of obesity WHO grade ≥ 1 of 44.5%, a rate of diabetes mellitus type II of 14.8%, and a rate of oral anticoagulants of 14.3% (Table 1).

**Table 1:** Baseline characteristics of the study population

	All = 182 (100%)
Sex	
Male, *n* (%)	108 (59.3)
Female, *n* (%)	74 (40.7)
Age at operation [years] mean value (MV) (range)	59.5 (21–84)
CCI median MV (±SD)	3.41 (±2.4)
Diabetes mellitus, *n* (%)	27 (14.8)
Anticoagulation, *n* (%)	26 (14.3)
Immunosuppression, *n* (%)	17 (9.3)
History of malignancy, *n* (%)	61 (33.5)
Smoking, *n* (%)	35 (19.2)
Operation due to recurrent incisional hernia	3 (1.6)
Obesity WHO grade	
0	101 (55.5)
1	44 (24.2)
2	26 (14.3)
3	11 (6)

### High CPSP rate and clinical data with no impact on CPSP

According to the criteria described in materials and methods the presence of CPSP was assessed among the 182 patients. This revealed an overall rate of 54.4% of patients suffering from CPSP. Of note, 14.8% of the patients were identified to suffer from severe CPSP (sCPSP) after open incisional hernia surgery and the rate of moderate CPSP (modCPSP) rate was 39.6%. No significant differences in gender, age, CCI were identified between the patient cohort with CPSP and the patient cohort without pain. Similarly, operation time, mesh type (heavy weight or light-weight mesh), and transfascial fixation did not differ significantly between both patient groups.

The complexity of hernias also was not different, either. There were also no significant differences between the two groups in the postoperative hemoglobin trend (difference between the value preoperatively and the value at discharge), the length of hospital stay (*p* = 0.172), the low rate of recurrence of the hernia (1.2% vs. 1%; *p* = 0.900), and complications ≥ 3 (24.1% vs. 14.1%; *p* = 0.086) according to the Clavien–Dindo classification (Table 2).

**Table 2:** Clinical data comparing “no CPSP” and “CPSP”

	No CPSP = 83 (45.6%)	CPSP = 99 (54.4%)	*p *value
Baseline patient characteristics			
Sex			0.704
Male *n* (%)	48 (57.8)	60 (60.6)	
Female *n* (%)	35 (42.2)	39 (40.7)	
Age at operation [years] mean value (MV) (range)	61.2 (24–81)	58.1 (21–84)	0.103
CCI median MV (SD)	3.70 (2.6)	3.17 (2.3)	0.147
Pre-existing conditions			
Diabetes mellitus *n* (%)	11 (13.3)	16 (16.2)	0.582
Anticoagulation *n* (%)	13 (15.7)	13 (13.1)	0.627
Immunosuppression *n* (%)	10 (12)	7 (7.1)	0.250
History of malignancy *n* (%)	31 (37.3)	30 (30.3)	0.316
Smoking *n* (%)	15 (18.1)	20 (20.2)	0.717
Obesity WHO grade			0.455
0	47 (56.6)	54 (55.5)	
1	16 (19.3)	28 (28.3)	
2	14 (16.9)	12 (12.1)	
3	6 (7.2)	5 (5.1)	
Perioperative information			
Hernia, *n* (%)			0.521
Complex	43 (51.8)	56 (56.6)	
Non-complex	40 (48.2)	43 (43.4)	
Mesh, *n* (%)			0.925
Heavy weight	34 (43)	42 (43.8)	
Light weight	45 (57)	54 (56.3)	
Operating time [min] MV (SD)	130.3 (58.6)	134.0 (57.8)	0.673
Hemoglobin difference [mg/dl] MV (SD)	− 2.81 (2.3)	− 2.35 (1.5)	0.126
Transfascial fixation *n* (%)	71 (88.8)	86 (90.5)	0.700
Complications *n* (%)			
Clavien–Dindo > 2	20 (24.1)	14 (14.1)	0.086
Bleeding	1 (1.2)	2 (2)	0.674
Days in hospital MV (SD)	8.7 (5.2)	7.8 (3.4)	0.172
Hernia recurrence *n* (%)	1 (1.2)	1 (1%)	0.900

### Analgesics score as a predictor of CPSP

**Fig. 2:**
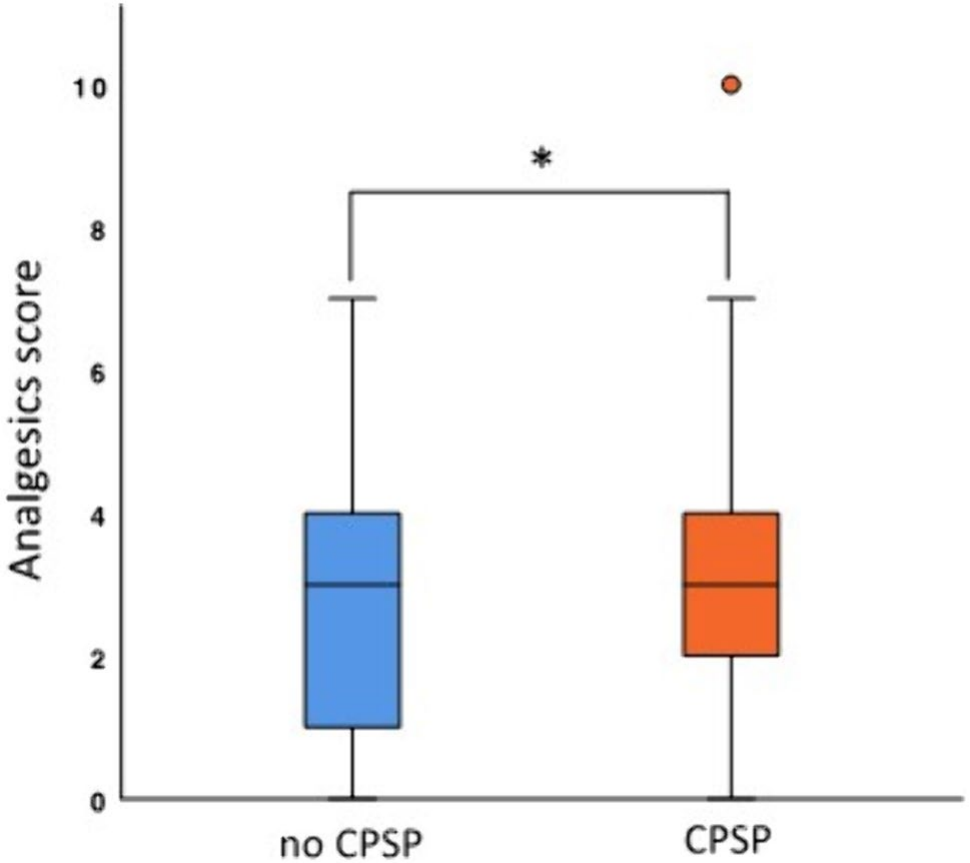
Analgesics score as a predictor of CPSP

Patients who developed a CPSP showed a significantly higher analgesic medication on the day of discharge to pain relief (*p* = 0.047) using the analgesics score. For patients with CPSP, the mean score was 3.35 (± 1.7), and 2.86 (± 1.6) for those without CPSP (Fig. 2).

### Scoring of the EuraHS QoL questionnaire

**Fig. 3:**
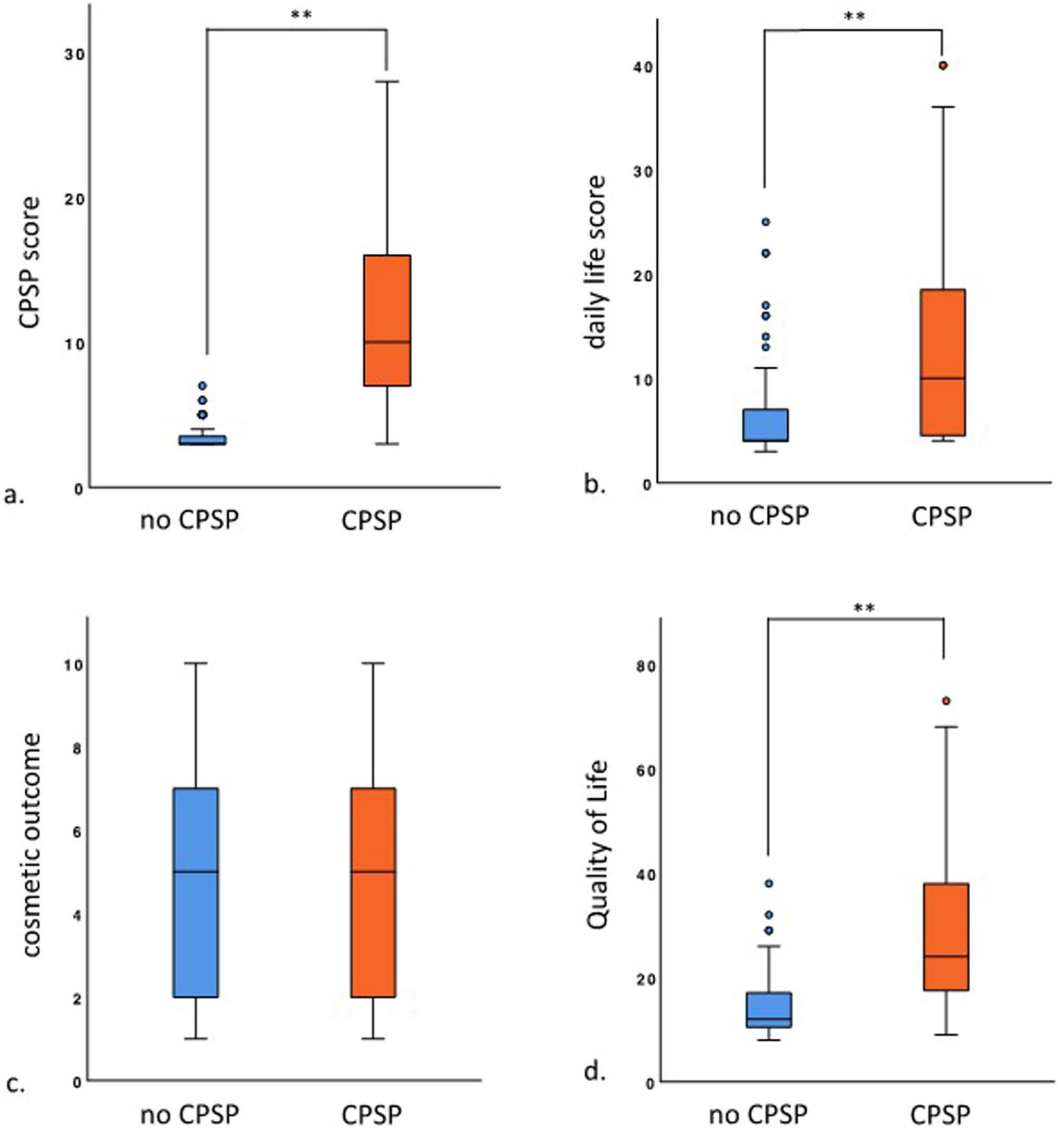
Influence of CPSP on quality of life

Patients with CPSP were found to have a significantly reduced quality of life (14.5 vs. 29.3; *p* < 0.001) according to the EuraHS QoL questionnaire. The pain category (CPSP score) in the questionnaire showed a significantly higher score in patients with CPSP (3.5 vs. 11.5; *p* < 0.001). Similarly, the category limitation in daily life, activities, and work (daily-life score) was significantly higher in patients with CPSP (6.4 vs. 12.9; *p* < 0.001). Only the cosmetic outcome showed no differences between the two groups (4.7 vs. 4.9; *p* = 0.667) underlining the overall contribution of the pain component and the reduction of daily-life activities component to the augmented scores (Fig. 3).

**Fig. 4:**
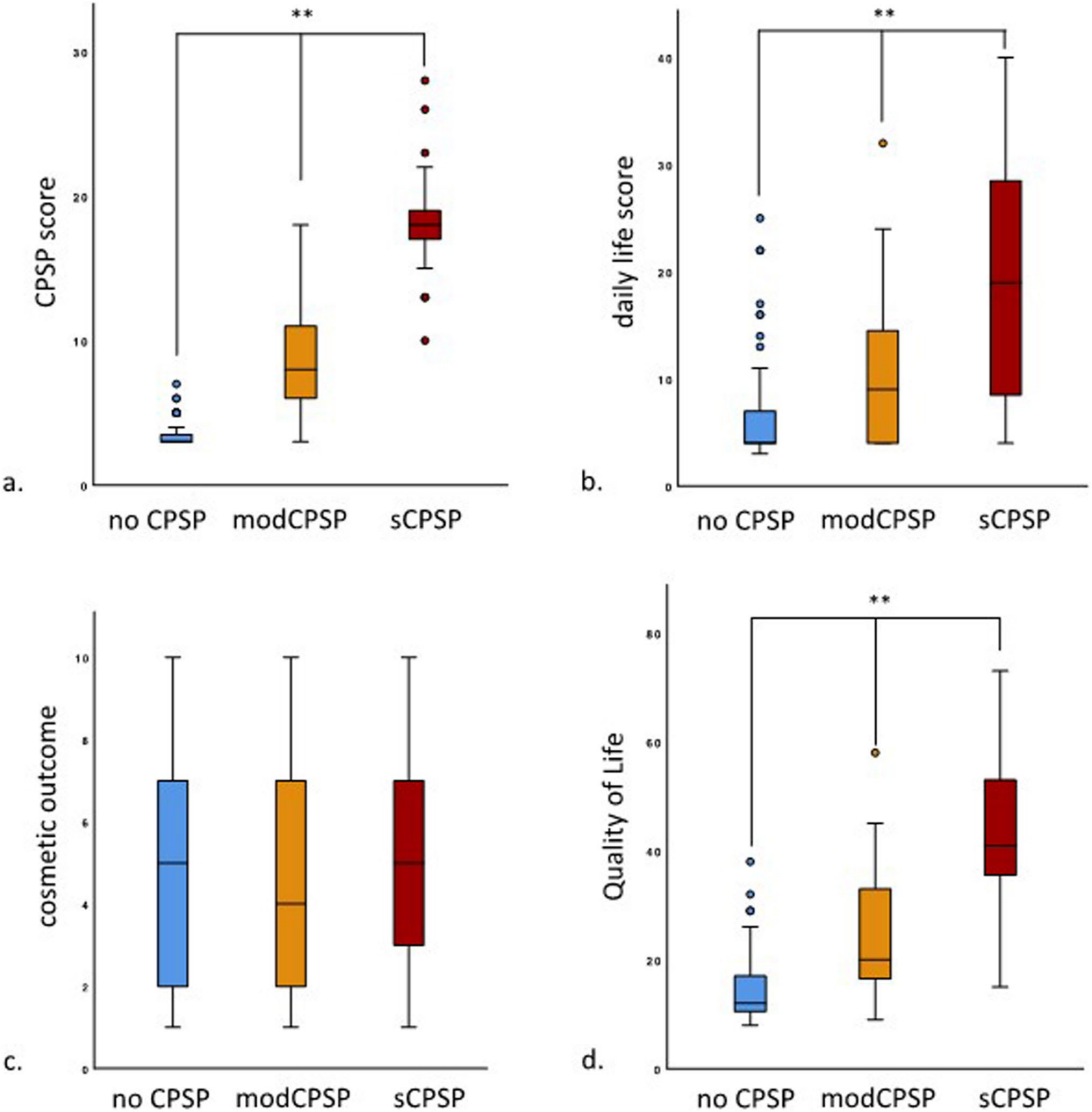
Subgroup analysis of complex hernia

The subgroup analysis between the three groups, no CPSP (*n* = 83), modPSP (*n* = 72), and sCPSP (*n* = 27), showed a significantly difference in the “CPSP score” (3.5 vs. 9.0 vs. 18.3; *p* < 0.001), the “daily-life score” (6.4 vs. 10.7 vs. 18.9; *p* < 0.001), and the quality of life (14.5 vs. 24.3 vs. 42.7; *p* < 0.001). The cosmetic outcome has no significantly differences between the three groups (4.7 vs. 4.7 vs. 5.5; *p* = 0.401) (Fig. 4).

### Subgroup complex hernia had longer operating time with no impact on analgesics score

In a subgroup analysis between patients with complex and non-complex hernias regarding perioperative data and the EuraHS QoL questionnaire only a longer operation time was found in complex hernias (147.6 min vs. 114.1 min; *p* = 0.001). The other clinical data, the analgesics score, and the scoring of the EuraHS QoL questionnaire showed no significant differences between the complex and non-complex hernias (Table 3).

**Table 3:** Subgroup: complex hernias. Perioperative information and EuraHS scoring

	Complex hernia = 99 (54.4%)	Non-complex hernia = 83 (45.6%)	*p* value
Perioperative information			
Mesh *n* (%)			0.081
Heavy weight	36 (36.4%)	40 (48.2%)	
Light weight	60 (60.6%)	39 (47%)	
Others	3 (3%)	4 (4.8%)	
Operating time [min] MV (SD)	147.6 (51.9)	114.1 (60)	0.001
Hemoglobin difference [mg/dl] MV (SD)	− 2.74 (2.0)	− 2.29 (1.7)	0.141
Analgesics score median MV (SD)	3.28 (1.6)	2.94 (1.8)	0.172
Days in hospital median [days] MV (SD)	8.8 (4.2)	7.5 (4.4)	0.052
Complications *n* (%)			
Clavien–Dindo > 2	21 (21.2)	13 (15.7)	0.339
Bleeding	2 (2)	1 (1.2)	0.674
Scoring of the EuraHS QoL questionnaire			
CPSP score MV (SD)	7.1 (5.2)	8.5 (6.1)	0.120
Daily-life score MV (SD)	9.6 (7.9)	10.2 (8.1)	0.591
Cosmetic outcome MV (SD)	4.6 (3.0)	4.9 (2.9)	0.488

## Discussion

In this study, the incidence of more than 50% CPSP following open incisional hernia points to a currently underestimated problem in this cohort of patients. Even though the patients in our cohort were adequately treated surgically with an overall low morbidity and low hernia recurrence after 2 years, more than half of the patients report discomfort and pain following open incisional hernia repair.

We hypothesized that comorbidities or postoperative complications might influence CPSP. We, therefore, determined the CCI index in our patients. The CCI index is an established method for classifying comorbidities that can change the risk of death and allows to compare comorbidities in different patient cohorts [15]. Using the CCI calculation, the average 10-year mortality in our patient cohort is very high and is estimated to be 52%. Additionally, further known risk factors for postoperative wound infections, such as obesity and anticoagulation therapy, are present in a high number of patients in our cohort. Even though our study population shows a high CCI, neither the CCI index nor postoperative complications did correlate with the occurrence of CPSP.

Chronic postoperative pain occurs 3–4 times more frequently after incisional hernia repair than after inguinal hernia repair (10–14% of patients) [14, 21]. This suggests that either previous surgical trauma, or anatomic differences between the abdominal wall and the inguinal region may contribute to the higher rates of CPSP compared to CPIP. Patients with CPSP also suffer more from a reduced quality of life after surgical incisional hernia repair than CPIP patients. Most surgeons report a recurrence rate up to 10–20% after incisional hernia repair [22, 23]. In our cohort, the clinical recurrence rate was very low (1% vs. 1.2%). However, it should be noted that the follow-up in our study might be too short to detect all recurrent hernia.

Previous studies have shown a higher risk of CPSP in patients with complex hernias [24–26]. In the comparison of complex versus non-complex hernias in our cohort, we did not find any differences in CPSP risk and the analgesics score. However, this conclusion might be biased due to the heterogeneity of the population in our cohort.

Although CPSP is observed frequently in everyday life, it is has been poorly documented. So far, only short-term outcomes (for example, 3 months postoperatively) and other complications (infections, recurrences) have been studied [27]. Through our retrospective analysis of real-world data from a non-selected cohort, we identified predictors contributing to the development of CPSP. We examined outcomes of patients with and without CPSP. Our study population reflects the current situation of care in clinical practice in a tertiary center and shows a CPSP rate of 54.4% and with 14.8% severe CPSP. In the few known studies, rates between 26.2 and 39% were reported [4, 6, 7]. This illustrates that CPSP is an insufficiently researched but nevertheless frequent and underestimated problem after incisional hernia repair. Thus, in our cohort, almost every second patient is affected by chronic pain, which in turn contributes to a significant reduction in quality of life. This relationship is a well-known and well-understood problem, which has already been clearly illustrated in numerous studies [28].

Few studies have been conducted on the therapeutic management of CPSP. The focus of therapy has been on the pharmacotherapy of pain. Since patients with CPSP often have a neuropathic pain component, most recommendations for therapy are based on the treatment of known neuropathic pain. For example, anticonvulsants, tricyclic antidepressants or topical applications (lidocaine, capsaicin) form the basis of therapy [29, 30]. In addition, conventional analgesics are used depending on the severity of the symptoms. Non-drug therapy options such as interventions on nerves (nerve blocks), psychological treatment options but also lifestyle modifications are discussed to improve the pain symptoms. To date, there is no gold standard, meaning that the treatment of CPSP must be individually tailored and adapted to each patient [31]. The most important known aspect in the treatment of CPSP to date is good perioperative pain management [32]. To be able to offer better treatment management in the future, it is important to identify patients at risk and to treat perioperative pain adequately.

The evaluation of the EuraHs QoL questionnaire with the newly defined “CPSP score” and the “daily-life score” showed a significant difference between the patient group with and without CPSP. Patients with CPSP show significantly more pain on the one hand and increased restrictions in everyday life, movement, and work on the other. This severely restricts their quality of life. The current therapeutic approach to prevent chronic pain and to improve quality of life is adequate analgesia, which should be started immediately after surgery and continuously adjusted [33]. By understanding the pathophysiology of chronic pain, further studies could generate other therapeutic approaches in the future.

We identified the analgesics score as a significant predictor on the development of a CPSP in our cohort. The analgesic score is a good measurement tool to relate pain and the necessary pain medication to avoid a misjudgement if, for example, there is no pain (NRS < 3) but a high need for opioids. Patients with CPSP require more analgesics to be pain free (NRS < 3) than patients without CPSP. A high analgesia requirement during hospitalization may already indicate a developing CPSP. The use of the analgesics score could already be proven in a retrospective study as an influencing factor on the development of CPSP pain after inguinal hernia surgery (CPIP) [14]. Since a significant influence is now also shown for incisional hernias, this score could possibly indicate chronic pain in general after hernia surgery. However, this assumption and the detailed pathogenesis behind the development of CPSP should be validated in a prospective study. In a previous study, evidence was found that suggests that the adaptive immune system may play a role in the development of chronic postoperative pain [34]. One question that still needs to be clarified is that of optimal postoperative analgesia in the prevention of chronic pain; further studies are needed here to genetically screen at-risk patients in the future to ensure optimal analgesia and metabolization of pain medication [35]. Many questions remain unanswered.

Taken together, we demonstrate that CPSP is a relevant problem following open incisional hernia repair. This raises the question whether the treatment of incisional hernias with open herniotomy is beneficial for the patient, or if new minimal invasive techniques, e.g., robotic-assisted surgery should be considered. Nevertheless, with the knowledge about CPSP, an early identification of potential CPSP patients, a differentiated indication as well as individual risk–benefit consideration for surgery should be of absolutely crucial importance [36].

### Strengths and limitations

The strength of this study is its adequately power, as our data reflect everyday clinical care in hospitals. The size of the selected collective allows a representation of real conditions and, thus, reliable answers to the questions. The major limitations of our study are its retrospective nature and single-center design, and the limited number of the risk factors that were investigated.

## Conclusion

Since there are few studies CPSP after incisional hernia repair, the aim of this study was to delineate the relevance, identify predictors of its development in our patients and to allow early identification of patients at higher risk for CPSP. In summary, in our collective, having an elevated analgesics score to be equated with having more postsurgical pain is a predictor of developing CPSP. This score may help to improve the identification of potential CPSP patients in the future. However, further studies to clarify pathogenesis and confirm predictors are needed to improve therapeutic approaches.
